# Effects of long-term reducing gastroplasty roux-y on body weight and clinical metabolic comorbidities in a bariatric surgery service of a university hospital

**DOI:** 10.1186/1758-5996-7-S1-A154

**Published:** 2015-11-11

**Authors:** Cátia Ferreira da Silva, Larissa Cohen, Luciana d'Abreu Sarmento, Felipe Monnerat Marino Rosa, Eliane Lopes Rosado, João Régis Ivar Carneiro, Antônio Augusto Peixoto de Souza, Fernanda Cristina Carvalho Mattos Magno

**Affiliations:** 1 Private Clinic, Rio de Janeiro, Brazil

## Background

Obesity is a major public health issue in Brazil and in the world, increasing the rate of mortality due to comorbidities like: type-2 diabetes mellitus (DM2), arterial hypertension (AH), dyslipidemias, among others. Conventional obesity treatments show little effect in the long term, leading to an increase in the search for bariatric surgery as an alternative for the control and healing of comorbidities.

## Objective

To evaluate type-2 diabetes mellitus, arterial hypertension and dyslipidemia in patients submitted to Roux-En-Y Gastric Bypass Surgery (RYGB) in the late post-operative period.

## Materials and Methods

Retrospective analysis of 59 patients from PROCIBA (bariatric surgery program of a University Hospital at Rio de Janeiro). Anthropometric (Height and Corporal Weight) and laboratorial (LDL, HDL, VLDL, Triglycerides (TG) and Glucose) data at pre-operative and post-operative periods accessed over medical records. Data comparison was conducted through ANOVA post-hoc Bonferroni test for anthropometric data and paired T-test for laboratorial data. A value of p<0.05 was considered as significant.

## Results

**Figure 1:**
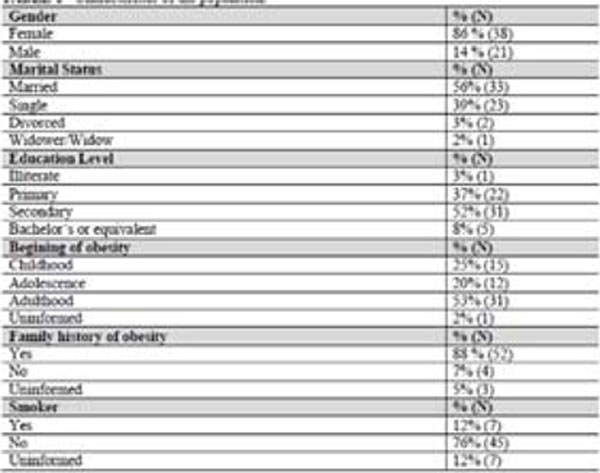
Characteristics of the population

**Figure 2:**
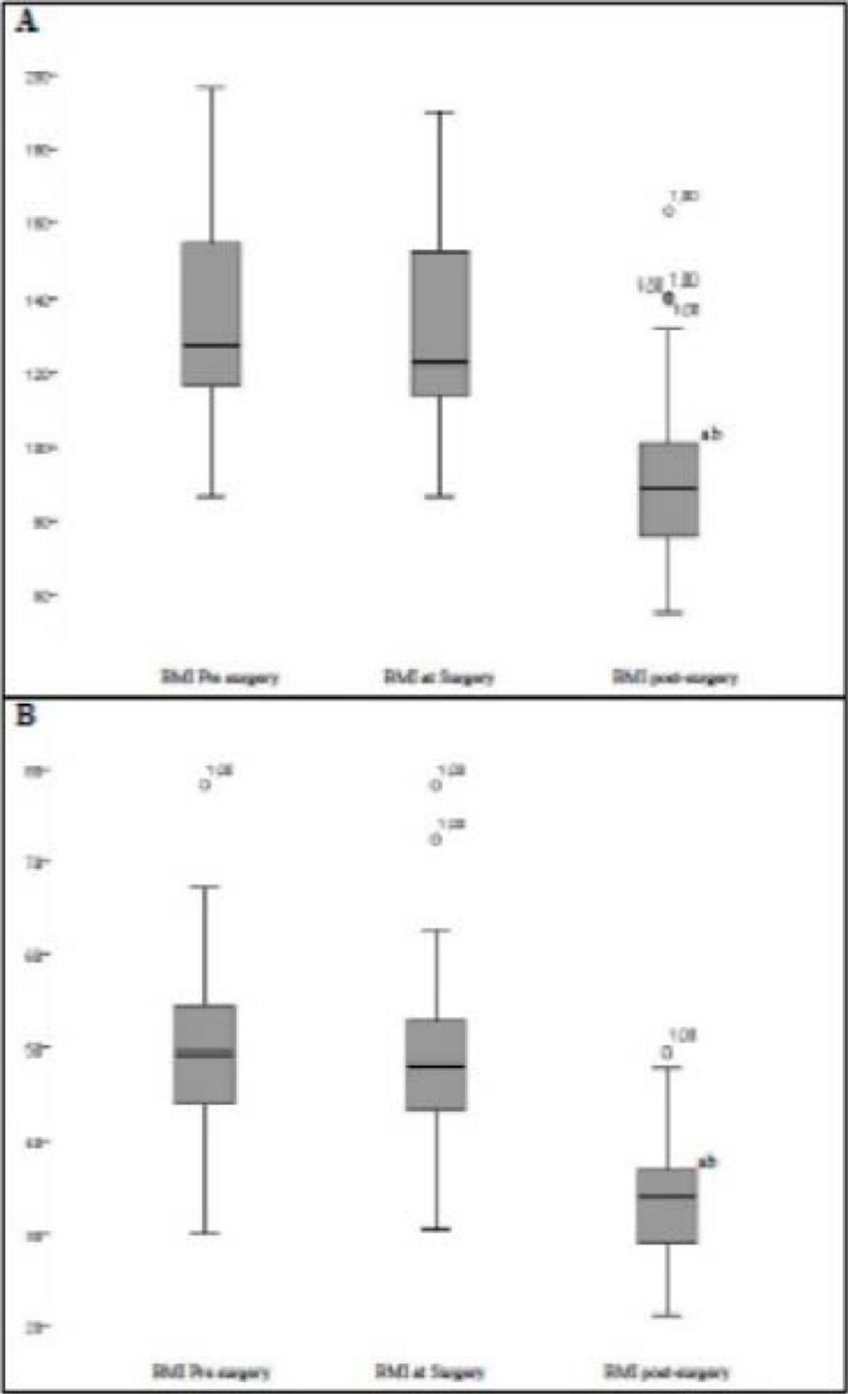
Assessment of body weight (A) and BMI (B) prior to surgery, on the day of surgery and after the procedure. ANOVA for repeated measure with post hoc Bonferroni and significance level 0.05 where: a = p <0.05 vs. weight preoperatively and b = p <0.05 vs. weight the day of surgery.

**Figure 3:**
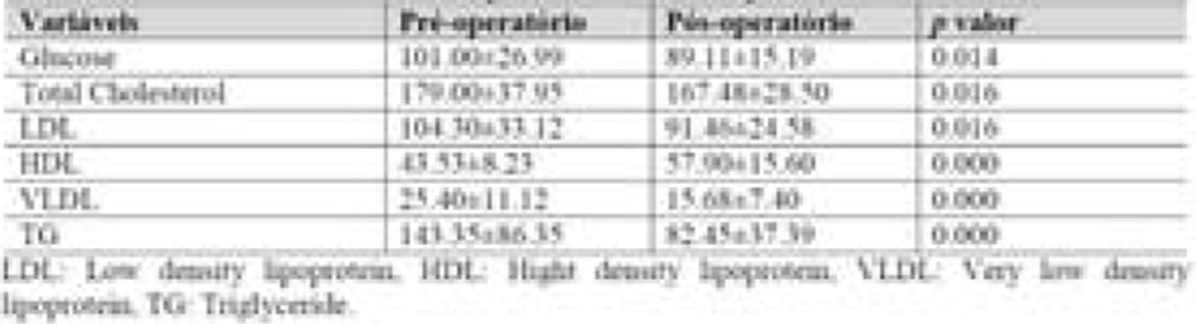
Evaluation of glucose and lipemia pre and post-operative.

83% of patients were female, with a mean age of 43±11 yrs.-old and 52% had completed high school education (Figure 1). Post-operative mean time was 7±3 yrs. Weight and body mass index (BMI) reduction were registered post-operatively (133±24 kg vs 91±22 kg and 49±8 kg/m2 vs 33±6 kg/m2, respectively, p<0.05) (Figure 2). Lower laboratory blood test values were registered post-operatively for glucose (101.00±26.99 vs 89,11±15.19, p=0.014), total cholesterol (179.00±37.95 vs 167.48±28.50, p < 0.016), LDL (104.30±33.12 vs 91.46±24.58, p=0.016), VLDL (25.40±11.12 vs 15.68±7.40, p< 0.01), TG (143.35±86,35 vs 82.45±37.39, p < 0.01), although higher HDL levels was registered (43.53±8.23 vs 57.90±15.60, p <0.01 ) (table 2). Prevalence in the pre-operative period for AH, DM2 and dyslipidemia were 76%, 36% and 27%, respectively. At the end of this study, 40% of patients were still in treatment for SAH (Figure 3). Remission for diabetes and dyslipidemia was registered in 81% and 94% patients, respectively (Figure 3).

## Conclusion

RYGB has shown an effective procedure in the long term, leading to weight loss and remission for DM2 and dyslipidemia.

